# Anti-SARS-CoV-2 vaccination in people with multiple sclerosis: Lessons learnt a year in

**DOI:** 10.3389/fimmu.2022.1045101

**Published:** 2022-10-17

**Authors:** Maura Pugliatti, Hans-Peter Hartung, Celia Oreja-Guevara, Carlo Pozzilli, Laura Airas, Mona Alkhawajah, Nikolaos Grigoriadis, Melinda Magyari, Bart Van Wijmeersch, Magd Zakaria, Ralf Linker, Andrew Chan, Patrick Vermersch, Thomas Berger

**Affiliations:** ^1^ Department of Neuroscience and Rehabilitation, University of Ferrara, Ferrara, Italy; ^2^ Interdepartmental Center of Research for Multiple Sclerosis and Neuro-inflammatory and Degenerative Diseases, University of Ferrara, Ferrara, Italy; ^3^ Department of Neurology, Medical Faculty, Heinrich-Heine-University Düsseldorf, Düsseldorf, Germany; ^4^ Brain and Mind Center, University of Sydney, Sydney, NSW, Australia; ^5^ Department of Neurology, Palacky University Olomouc, Olomouc, Czechia; ^6^ Department of Neurology, Hospital Clínico San Carlos, Instituto de Investigación Sanitaria del Hospital Clínico San Carlos (IdISSC), Madrid, Spain; ^7^ Faculty of Medicine, Complutense University of Madrid (UCM), Madrid, Spain; ^8^ Multiple Sclerosis Center, S. Andrea Hospital, Department of Human Neuroscience, University Sapienza, Rome, Italy; ^9^ Division of Clinical Neurosciences, University of Turku, Turku, Finland; ^10^ Neurocenter of Turku University Hospital, Turku, Finland; ^11^ Section of Neurology, Neurosciences Center, King Faisal Specialist Hospital and Research Center, Riyadh, Saudi Arabia; ^12^ College of Medicine, Al Faisal University, Riyadh, Saudi Arabia; ^13^ Laboratory of Experimental Neurology and Neuroimmunology, Second Department of Neurology, American Hellenic Educational Progressive Association (AHEPA) University Hospital, Aristotle University of Thessaloniki, Thessaloniki, Greece; ^14^ Danish Multiple Sclerosis Center, Department of Neurology, Rigshospitalet, Copenhagen University Hospital, Copenhagen, Denmark; ^15^ Universitair Multiple Sclerosis (MS) Centrum, Hasselt-Pelt, Belgium; ^16^ Revalidatie & Multiple Sclerosis (MS), Noorderhart, Pelt, Belgium; ^17^ Rehabilitation Research Center (REVAL) & Biomedical Research Institute (BIOMED), Hasselt University, Hasselt, Belgium; ^18^ Department of Neurology, Ain Shams University, Cairo, Egypt; ^19^ Clinic and Polyclinic for Neurology, Universitätsklinikum Regensburg, Regensburg, Germany; ^20^ Department of Neurology, Inselspital Bern, Bern University Hospital, University of Bern, Bern, Switzerland; ^21^ University of Lille, Inserm U1172 LilNCog, CHU Lille, FHU Precise, Lille, France; ^22^ Department of Neurology, Medical University of Vienna, Vienna, Austria; ^23^ Comprehensive Center for Clinical Neurosciences and Mental Health, Medical University of Vienna, Vienna, Austria

**Keywords:** COVID-19, vaccines, SARS-CoV-2, multiple sclerosis, disease modifying therapies, immune response, adverse events

## Abstract

It has been over a year since people with multiple sclerosis (pwMS) have been receiving vaccines against severe acute respiratory syndrome coronavirus 2 (SARS-CoV-2). With a negligible number of cases in which vaccination led to a relapse or new onset MS, experts around the world agree that the potential consequences of COVID-19 in pwMS by far outweigh the risks of vaccination. This article reviews the currently available types of anti-SARS-CoV-2 vaccines and the immune responses they elicit in pwMS treated with different DMTs. Findings to date highlight the importance of vaccine timing in relation to DMT dosing to maximize protection, and of encouraging pwMS to get booster doses when offered.

## Introduction

Since the outbreak of severe acute respiratory syndrome coronavirus 2 (SARS-CoV-2) was first reported in Wuhan China on 31 December 2019, it has caused over 6 million deaths (1).

The development of vaccines against SARS-CoV-2 started as soon as the genetic sequence of the virus was made publicly available in January 2020 and has progressed at lightning speed thanks to previous knowledge of other coronaviruses, advances in vaccine design and unprecedented global funding.

**Figure 1:**
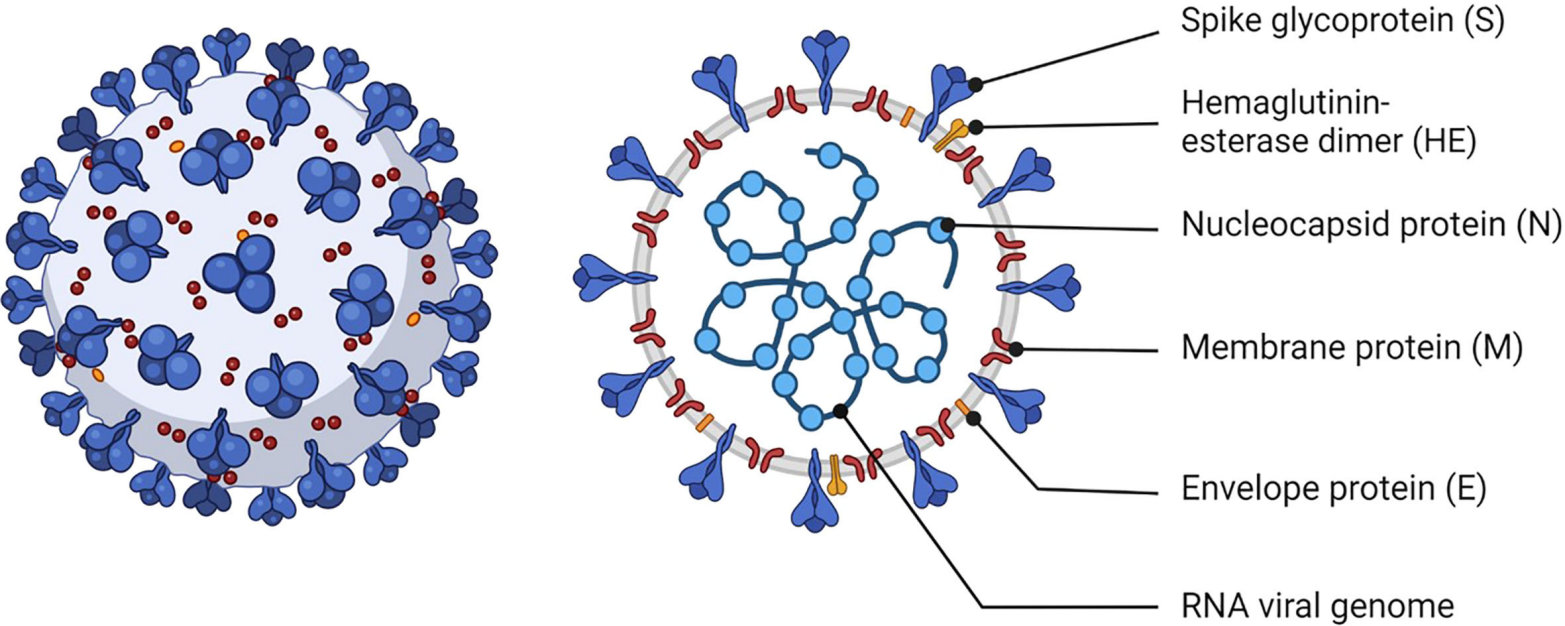
Schematic of SARS-CoV-2. The viral RNA is contained within the viral envelope, a lipid bilayer derived from the host cell membrane. Structural proteins such as spike (S), membrane (M), envelope (E) proteins are embedded in the viral envelope. The S protein is essential for the virus to attach to and enter uninfected cells. Created with BioRender.com.

SARS-CoV-2 is a single-stranded positive-sense RNA virus (**Figure 1**). Spike (S) proteins on the virion membrane mediate entry into host cells by binding to angiotensin-converting enzyme 2 (ACE2) and triggering membrane fusion (2, 3). S proteins have been the main target of vaccines since antibodies against this protein block virus entry to host cells and inhibit viral replication (4).

As of September 2022, six anti-SARS-CoV-2 vaccines have been authorized for use in the European Union by the European Medicines Agency (EMA) (5). These vaccines reduce the risk of severe disease by activating humoral and cellular immune responses against SARS-CoV-2 (6, 7).

Multiple sclerosis (MS) is a chronic immune-mediated demyelinating disease of the central nervous system (CNS) that causes significant and irreversible neurological disability. People with MS (pwMS) are not at greater risk of SARS-CoV-2 infection than the general population (8). However older age, male sex, comorbidities, a high Expanded Disability Status Scale score, and treatment with anti-CD20 monoclonal antibodies or high dose glucocorticosteroids, are risk factors for a severe SARS-CoV-2-related disease (COVID-19) course (9–12), defined by acute respiratory distress syndrome, intensive care unit admission and death.

Some disease modifying therapies (DMTs) for MS exert their effects on humoral and cellular immune activity and, thus, may affect the response to anti-SARS-CoV-2 vaccines. This review examines the effects of different DMTs on the response to anti-SARS-CoV-2 vaccines in pwMS and the recommended timing of vaccination relative to DMT dosing to achieve maximum vaccine efficacy.

## Vaccination in pwMS: General considerations

Systemic infections can worsen MS, thus, vaccination will lower the risk of relapses by reducing the risk of infections (13). Nevertheless, there are still some concerns about the safety and efficacy of vaccines in pwMS.

There is no significant evidence of a causal relationship between the onset or deterioration of MS and vaccination against hepatitis B virus, human papillomavirus, seasonal influenza, measles-mumps-rubella, variola, tetanus, Bacillus Calmette–Guérin (BCG), polio, typhoid fever, or diphtheria (14, 15). One study suggested there may be an increased relapse rate in travelers with MS following vaccination with live-attenuated yellow fever vaccines (16).

Live attenuated vaccines are contraindicated in pwMS on immunosuppressive treatments because of the risk of infection. Inactive vaccines may be less effective in pwMS on immunosuppressive treatments as they can inhibit the development of a protective immune response.

Because the safety and efficacy of anti-SARS-CoV-2 vaccines in pwMS is still being assessed, vaccination guidance should take into consideration the effects of any MS therapy on the immune system, the type of vaccine, disease burden and risk of infection.

## Types of anti-SARS-CoV-2 vaccines

Currently approved vaccines in the EU are either RNA vaccines (Moderna and Pfizer/BioNTech vaccine), viral vector vaccines (Oxford/AstraZeneca and Janssen), protein vaccines (Novavax) or whole virus, inactivated, adjuvanted (Valneva).

RNA vaccines deliver the mRNA of the SARS-CoV-2 virus S protein, so that it is endogenously expressed on cell surfaces (17). The immune system recognizes the protein as foreign triggering both cellular and humoral immunity. With this approach there is no risk of reversion to virulence or anti-vector immunity. RNA vaccines can be manufactured quickly and inexpensively so they can be rapidly deployed during emergencies, but they require storage at specific low temperatures (18). RNA vaccine candidates against Ebola and Zika are undergoing preclinical and clinical testing (19, 20).

The Pfizer/BioNTech vaccine (BNT162b2) and the Moderna vaccine (mRNA-1273) are administered intramuscularly, two doses are required at least 21 or 28 days apart, respectively (21, 22).

Viral vector vaccines use an unrelated harmless adenovirus (the viral vector) to deliver the SARS-CoV-2 S protein gene. Host cells use the genetic material to produce the specific viral protein, which triggers a cellular and humoral immune response.

The Oxford/AstraZeneca vaccine (ChAdOx1 nCoV-19) requires two intramuscular injections given 4-12 weeks apart (23). The Janssen COVID-19 vaccine (Ad26.COV2-S) requires only one dose, and a booster can be given at least 2 months after the primary dose (24).

Novavax is a protein vaccine that contains the full-length SARS-CoV-2 S protein and a Matrix-M1 adjuvant to boost the immune response (25). Two doses should be administered intramuscularly with an interval of 3-4 weeks.

The latest vaccine to be approved by the EMA in June 2022, Valneva, contains whole particles of the original strain of SARS-CoV-2 that have been inactivated and cannot cause the disease. Two intramuscular injections 4 weeks apart are required for protection.

At the time of writing, over 85 vaccine candidates are in Phase 3 clinical trials (26). These include inactivated vaccines (Sinopharm, Sinovac), live-attenuated vaccines (Meissa) and DNA-based vaccines (Inovio) (27–30). Inactivated and live-attenuated anti-SARS-CoV-2 vaccines are unlikely to be used in pwMS.

Because most published data in pwMS are with the mRNA-1273, BNT162b2 vaccine and ChAdOx1 nCoV-19 vaccines, this article will focus on these.

Most studies to date indicate that these vaccines do not increase the risk of relapse activity or prevent DMTs from being fully effective (31–33). There have been a series of clinical cases in which a temporal association between vaccine administration and MS relapses have been reported (34–37). Although this association is rare, it might be an adverse event of anti-SARS-CoV-2 vaccination that will need to be examined further (see ‘Vaccination and adverse events’ section for more details).

## Anti-SARS-CoV-2 vaccination, DMTs and immune response

Most national neurology associations and organizations agree on not discontinuing MS treatment with DMTs during the pandemic, even during active infection (38–41). However, to maximize the effectiveness of anti-SARS-CoV-2 vaccines the mode of action of DMTs and the timing of immunization should be considered (**Table 1**).

**Table 1:** DMT, vaccination response and timing recommendations.

DMT	Vaccine	Timing	Response	Other
Beta interferons	mRNA-1273 BNT162b2 vaccine ChAdOx1 nCoV-19	No adjustment required	Comparable to untreated MS patients and healthy controls	Less likely to experience short term reactogenicity to vaccine
Glatiramer acetate			
Dimethyl fumarate			
Teriflunomide				
Natalizumab				
Sphingosine 1-phosphate receptor modulators		No adjustment required	Low humoral response in many patients	In new starters anti-SARS-CoV-2 vaccination should be completed at least 4 weeks before Less likely to experience short term reactogenicity to vaccine
Anti-CD20 based treatments		Vaccination should take place 5–6 months after the last anti-CD20 treatment	Low humoral response	In new starters anti-SARS-CoV-2 vaccination should be completed at least 6 weeks before
Cladribine		Vaccination should take place 2-4 weeks before course of therapy	Mostly comparable to healthy controls	In cases of lymphopenia, it is advisable to wait until recovery of lymphocyte count
Alemtuzumab		Vaccination should take place at least 3 months after the last course of therapy	Mostly comparable to healthy controls	In new starters anti-SARS-CoV-2 vaccination should be completed 4-6 weeks before.

Based on previous results from clinical trials and real-world experience exploring the response to other vaccines, as well as emerging data with anti-SARS-CoV-2 vaccines, no adjustments to vaccine administration are required in pwMS taking interferon-beta, glatiramer acetate, dimethyl fumarate, teriflunomide or natalizumab (42–47). These DMTs do not affect the response to vaccinations in general, although in some cases they can cause lymphopenia which could interfere with immune response. To date, there are no reports that these DMTs impair the immune response to the mRNA-1273, BNT162b2 vaccine and ChAdOx1 nCoV-19 vaccines (48).

Similarly, no adjustments are required for pwMS already under treatment with fingolimod, ozanimod, ponesimod or siponimod. It has consistently been shown that most patients treated with S1P1 modulators exhibit low humoral and cellular immune responses to anti-SARS-CoV-2 vaccines, yet the likelihood of COVID-19 breakthrough disease with hospitalization is not increased (45, 49). These findings suggest that the mechanism of action of S1P1 modulators may offer protection against COVID-19 (for example, by reducing cytokine release in the CNS) (50, 51) and are driving further research into the contribution of T and B cell responses towards protection against SARS-CoV-2 (47). Ideally, in patients that are about to start treatment with sphingosine 1-phosphate receptor modulators, anti-SARS-CoV-2 vaccination should be completed at least 4 weeks before.

Because anti-CD20 based treatments can reduce the humoral immune response to some vaccines (52), patients about to start treatment with ocrelizumab and rituximab, are advised to complete anti-SARS-CoV-2 vaccination at least 6 weeks before infusion. If vaccination is not possible prior to treatment initiation, vaccine administration should be carefully planned to take place after B-cell repopulation. This could be achieved by administering the vaccine 5-6 months after the last anti-CD20 treatment (53).

Several studies have reported decreased humoral responses in patients treated with ocrelizumab or rituximab compared with healthy controls after anti-SARS-CoV-2 vaccination (46, 47, 49, 54–56). However, most MS patients treated with anti-CD20 monoclonal antibodies develop a cellular response after anti-SARS-CoV-2 vaccination, regardless of the low humoral response (57–60). Further studies are required to determine whether this translates into full protection against infection.

Most studies show that pwMS treated with cladribine or alemtuzumab develop a humoral response against S protein that is comparable to healthy controls (45, 49, 61, 62). Because cladribine tablets cause a rapid depletion of peripherally circulating B and T lymphocytes with a mean nadir at 13–24 weeks, it is advisable to complete vaccination against COVID-19 2-4 weeks before starting a course of treatment (63, 64). However, no adverse effects associated with vaccination have been found after treatment, so experts suggest that these patients should get the anti-SARS-CoV-2 vaccine when offered, unless they have a contraindication (65). In pwMS treated with alemtuzumab, it is recommended to complete vaccinations at least 6 weeks before starting treatment (63). If therapy has already started, patients should wait 3-6 months after the last dose for B cells to return to basal levels.

In patients taking high-dose steroids or who experience a clinical relapse, vaccination should be postponed by 4 weeks so that the response is more effective (31).

To develop protective immunity from anti-SARS-CoV-2 vaccines, pwMS receiving haematopoietic stem cell transplantation (HSCT) should wait at least 3 months after treatment before vaccination (66). Revaccination may be required in patients who were administered vaccines before autologous transplantation.

Continuous surveillance of vaccine effectiveness in pwMS taking immunomodulatory and immunosuppressive drugs is vital to inform future treatment strategies and vaccination protocols. At the time of writing there is consensus that a reduced response (cellular and/or humoral) to anti-SARS-CoV-2 vaccines is better than none and that the risks of COVID-19 by far outweigh any potential risks from the vaccine. Assessing patients’ serological status in post-vaccination check-ups will help to determine whether booster doses are required.

There are limited data about differences in effectiveness and safety between approved vaccines in pwMS. However, the CDC (Centers for Disease Control and Prevention) in the US, recommends using mRNA vaccines (mRNA-1273 and BNT162b2) over the Janssen vaccine Ad26.COV2-S in pwMS (67). One study showed that vaccination with mRNA-1273 resulted in a systematically 3·25-fold higher antibody level than with BNT162b2 vaccine (49), and another found a higher humoral response rate with the BNT162b2 vaccine compared to the inactivated vaccine, Sinovac, in MS patient cohorts (62).

## Vaccination and adverse events

The short-term COVID-19 vaccine reactions experienced by pwMS are similar to those reported in trials in the general population (31, 33, 68). The most common reactions being pain at injection site, fatigue, headache, and malaise (a general feeling of discomfort). Younger age, female sex and prior SARS-CoV-2 infection were associated with greater odds of experiencing adverse effects after vaccination. Interestingly, individuals treated with specific classes of DMT, such as sphingosine-1-phosphate receptor modulators or dimethyl fumarate, were less likely to experience short-term reactogenicity (33).

In July 2021, the EMA declared that myocarditis and pericarditis can occur in very rare cases after vaccination with anti-SARS-CoV-2 mRNA vaccines and recommended listing them as new side effects in the product information (69). A US study showed that the risk of myocarditis was highest after the second vaccination dose in adolescent males and young men (70). There are no reports on the incidence of myocarditis and pericarditis after vaccination in pwMS.

Oxford/AstraZeneca’s viral vector vaccines have similar common side effects to the mRNA ones (feeling unwell, fatigue, fever, headache). In April 2021, the EMA reported a possible link between the Oxford/AstraZeneca vaccine and a very rare side effect of unusual blood clots in the brain (cerebral venous sinus thrombosis), the abdomen (splanchnic vein thrombosis) and in arteries combined with low levels of blood platelets (71), which led to updated guidance for healthcare professionals on how to minimise risks, as well as further advice on symptoms for vaccine recipients to look out for after vaccination. There is no indication that pwMS have a higher risk of blood clotting following vaccination.

It is still unclear whether anti-SARS-CoV-2 vaccination might induce an immunological response that could activate MS. Fever caused by vaccination can temporarily worsen MS symptoms, but there have been reports of longer-term disease worsening in some MS patients and a few cases of acute demyelinating disease onset.

For example, a 31-year-old Italian woman with stable MS (after a second cycle of cladribine) experienced a severe relapse 48 hours after receiving the 1st dose of the Pfizer/BioNTech vaccine (37). She made a full recovery after 5 days of treatment with methylprednisolone.

Four individuals aged 24 to 48 years experienced active demyelination in the optic nerve, brain, and/or spinal cord within 1-21 days of Moderna or Pfizer/BioNTech (1^st^ or 2^nd^ dose administration) (36).

There have also been reports of new onset of relapsing-remitting (RR) MS and new onset neuromyelitis optica (NMO) after vaccination (36). A 26-year-old white Hispanic woman showed optic neuritis, and new lesions in the brain and spinal cord 14 days after the 2^nd^ dose of Moderna vaccine, and a 33-year-old Caucasian man showed optic neuritis and new MRI lesions 1 day after the 2^nd^ dose of the Pfizer/BioNTech vaccine. Both recovered after treatment with methylprednisolone.

Described cases of new onset NMO include a 64-year-old Caucasian man who showed spinal syndrome 18 days after the 1^st^ dose of the Pfizer/BioNTech vaccine as well as extensive new MRI spinal cord lesions (36), and a 32-year-old male who presented with a 2-week history of acute confusional state and imbalance 1 week after receiving the 2^nd^ dose of the Sputnik vaccine (viral vector vaccine) (72). Both made a partial recovery after treatment.

Overall, the number of individuals who experience active CNS demyelinating disease is very small given the large number of pwMS who have received vaccination. Data to date suggest that a causal relationship between anti-SARS-CoV-2 vaccines and acute CNS demyelination is unlikely.

## ParadigMS Foundation experts’ consensus

Immunization against COVID-19 is highly recommended for all MS patients regardless of age and comorbidities. The vaccination course should be completed even if the first dose was associated with a temporary flaring of symptoms.

Family members should also be vaccinated in order to reduce risk and impact of infection in MS patients.

Because it takes up to 28 days after the first dose of the Pfizer-BioNTech vaccine and up to 22 days after the Oxford-AstraZeneca vaccine to reach some level of immunity, it is crucial to maintain precautions after initial vaccination.

Evidence of waning immunity 4-6 months after vaccination (73, 74), and the emergence of novel variants of concern that have the potential to cause increased disease severity and to decrease COVID-19 vaccine effectiveness, has led to several countries offering third vaccine doses or vaccine boosters, not just to the highest risk groups (including older and immunocompromised people) but to the general population by the end of 2021.

A third dose of the Pfizer-BioNTech or the Moderna vaccine has been shown to be safe and to significantly increase SARS-CoV-2 antibody levels in the general population (75, 76) and in pwMS (77, 78). Breakthrough infections in pwMS during the delta and omicron wave have been associated with low SARS-CoV-2 antibody levels, and a third vaccine dose significantly reduced the risk of infection during the Omicron wave (79).

At the time of writing several countries are offering fourth COVID-19 vaccine doses to people who are immunocompromised and care home residents as they have been shown to boost antibody levels and prevent severe omicron COVID-19 (80, 81).

Despite the recommendation that MS patients should take the offered vaccines, up to 20% are hesitant, mainly due to safety concerns (82–84). Factors such as younger age, low education level, lower perceived risk for COVID-19 infection, and higher functional disability have been independently associated with reduced vaccine willingness (83, 84). Consistent and context-specific vaccination counselling for pwMS will help tackle vaccine hesitancy and improve vaccine roll out in the most hesitant patient subgroups.

## Conclusions

The potential consequences of SARS-CoV-2 infection in MS patients outweigh the risks of vaccination.

Currently EU-approved anti-SARS-CoV-2 vaccines produce high immunogenicity associated with favorable safety profile in the MS population. Emerging data support not delaying vaccination or stopping MS treatment during the SARS-CoV-2 pandemic.

Clinicians should discuss anti-SARS-CoV-2 vaccination timing with pwMS to maximize the effectiveness of the vaccine, taking into consideration their risk of infection, the type of DMT they are taking, their current immune status, their general health and the coexistence of other diseases.

These recommendations will need to be regularly updated as knowledge of how pwMS respond to SARS-CoV-2 vaccines (and the extent to which they protect against new virus variants) is evolving very rapidly (85).

## Author contributions

TB, MP, H-PH led the conceptual framework of the manuscript and critically reviewed all versions of the article, read and approved the final manuscript, and agree to be responsible for all aspects of the work. CO-G, CP, LA, MA, NG, MM, BV, MZ, RL, AC, PV contributed to the conceptual development of the article, critically reviewed it, read and approved the final manuscript, and agree to be responsible for all aspects of the work. All authors contributed to the article and approved the submitted version.

## Funding

The authors acknowledge the financial and operational support of the ParadigMS Foundation that made it possible to produce this article. ParadigMS activities are co-funded by Sanofi, Roche and Merck.

## Acknowledgments

We would like to thank Monica Hoyos of Springer Healthcare Communications for medical writing assistance funded by ParadigMS Foundation. ParadigMS Foundation is a group of European, Middle Eastern and North African experts in multiple sclerosis. The content of this publication is based upon in-depth discussions on this topic by the group members at Expert Meetings. The current list of group members can be consulted at ParadigMS’s website.

## Conflict of interest

MP has received honoraria, consulting fees or travelling costs from Bayer, Biogen, Sanofi-Genzyme, Teva, Merck and Almirall. CO-G has received speaker and consultation fees from Biogen Idec, Celgene, Sanofi-Genzyme, Novartis, Roche, Merck and Teva. CP has served on scientific advisory boards for Novartis, Merck, Biogen, Bristol Myers Squibb, Roche, Janssen, Alexion, has received funding for travel and speaker honoraria from Merck, Biogen, Bristol Myers Squibb, Roche, Almirall, Janssen, Alexion and Novartis, and receives research support from Merck, Biogen, Novartis, Roche and Almirall. LA has received institutional research support from Novartis, Genzyme and Merck, and compensation for lectures and consulting fees from Novartis, Sanofi Genzyme, Merck, Biogen, Roche and Janssen. MA has received speaker honoraria, consulting fees, and/or educational travel support from Biogen, Merck, Sanofi-Genzyme, Roche, Novartis, Hikma and SAJA. NG has received honoraria and travel support, consultancy and lecture fees from Biogen Idec, Biologix, Novartis, Teva, Bayer, Merck Serono, Genesis Pharma, Sanofi – Genzyme, Roche, Elpen. He has also received research grants from Biogen Idec, Novartis, Teva, Merck Serono, Genesis Pharma, Sanofi – Genzyme and Roche. MM has served on scientific advisory boards for Sanofi, Novartis and Merck. She has received honoraria for lecturing from Biogen, Merck, Novartis, Roche, Genzyme and BMS. BW has received Research and Travel Grants, Honoraria for MS-Expert Advice and Speakers Fees from: Almirall, Actelion/Janssen, Bayer, Biogen, Celgene/BMS, Merck, Novartis, Roche, Sanofi-Genzyme and Teva. AC has received speakers’/board honoraria from Actelion (Janssen/J&J), Almirall, Bayer, Biogen, Celgene (BMS), Genzyme, Merck KGaA (Darmstadt, Germany), Novartis, Roche, and Teva, all for hospital research funds. He received research support from Biogen, Genzyme, and UCB, the European Union, and the Swiss National Foundation. PV has received honoraria and consulting fees from Biogen, Sanofi-Genzyme, Novartis, Teva, Merck, Roche, Imcyse, AB Science and BMS-Celgene. He also receives research support from Novartis, Sanofi-Genzyme and Roche. TB has received honoraria and consulting fees from Almirall, Bayer, Biogen, Biologix, Bionorica, Celgene/BMS, GSK, GW/Jazz Pharma, Horizon, MedDay, Merck, Novartis, Octapharma, Roche, Sandoz, Sanofi-Genzyme, TEVA, TG Therapeutics and UCB. He has received institutional research support from Almirall, Biogen, Bayer, Celgene/BMS, Merck, Novartis, Roche, Sanofi-Genzyme and Teva, and has participated in clinical trials sponsored by Alexion, Bayer, Biogen, Celgene/BMS, Merck, Novartis, Octapharma, Roche, Sanofi-Genzyme and Teva.

The remaining authors declare that the research was conducted in the absence of any commercial or financial relationships that could be constructed as a potential conflict of interest.

## Publisher’s note

All claims expressed in this article are solely those of the authors and do not necessarily represent those of their affiliated organizations, or those of the publisher, the editors and the reviewers. Any product that may be evaluated in this article, or claim that may be made by its manufacturer, is not guaranteed or endorsed by the publisher.

## References

[1] World Health Organization. WHO coronavirus (COVID-19) dashboard. Available at: https://covid19.who.int/ (Accessed July 30, 2022).

[2] Lan J Ge J Yu J Shan S Zhou H Fan S. Structure of the SARS-CoV-2 spike receptor-binding domain bound to the ACE2 receptor. Nature (2020) 581:215–20. doi: 10.1038/s41586-020-2180-5 32225176

[3] Jackson CB Farzan M Chen B Choe H. Mechanisms of SARS-CoV-2 entry into cells. Nat Rev Mol Cell Biol (2022) 23:3–20. doi: 10.1038/s41580-021-00418-x 34611326 PMC8491763

[4] Martínez-Flores D Zepeda-Cervantes J Cruz-Reséndiz A Aguirre-Sampieri S Sampieri A Vaca L. SARS-CoV-2 vaccines based on the spike glycoprotein and implications of new viral variants. Front Immunol (2021) 12:701501. doi: 10.3389/fimmu.2021.701501 34322129 PMC8311925

[5] European Medicines Agency. COVID-19 vaccines . Available at: https://www.ema.europa.eu/en/human-regulatory/overview/public-health-threats/coronavirus-disease-covid-19/treatments-vaccines/covid-19-vaccines (Accessed July 30, 2022).

[6] Ewer KJ Barrett JR Belij-Rammerstorfer S Sharpe H Makinson R Morter R. T Cell and antibody responses induced by a single dose of ChAdOx1 nCoV-19 (AZD1222) vaccine in a phase 1/2 clinical trial. Nat Med (2021) 27:270–8. doi: 10.1038/s41591-020-01194-5 33335323

[7] Almendro-Vázquez P Laguna-Goya R Ruiz-Ruigomez M Utrero-Rico A Lalueza A Maestro de la Calle G. Longitudinal dynamics of SARS-CoV-2-specific cellular and humoral immunity after natural infection or BNT162b2 vaccination. PloS Pathog (2021) 17 (12):e1010211. doi: 10.1371/journal.ppat.1010211 34962970 PMC8757952

[8] Chaudhry F Jageka C Levy PD Cerghet M Lisak RP. Review of the COVID-19 risk in multiple sclerosis. J Cell Immunol (2021) 3:68–77. doi: 10.33696/immunology.3.080 33959727 PMC8098748

[9] Sormani MP Schiavetti I Carmisciano L Cordioli C Filippi M Radaelli M. COVID-19 severity in multiple sclerosis: Putting data into context. Neurol Neuroimmunol Neuroinflamm (2021) 9:e1105. doi: 10.2139/ssrn.3884934 34753829 PMC8579249

[10] Etemadifar M Nouri H Maracy MR Akhavan Sigari A Salari M Blanco Y. Risk factors of severe COVID-19 in people with multiple sclerosis: A systematic review and meta-analysis. Rev Neurol (Paris) (2022) 178:121–8. doi: 10.1016/j.neurol.2021.10.003 PMC8566345 34836608

[11] Patel NJ D'Silva KM Hsu TY DiIorio M Fu X Cook C. Coronavirus disease 2019 outcomes among recipients of anti-CD20 monoclonal antibodies for immune-mediated diseases: A comparative cohort study. ACR Open Rheumatol (2022) 4:238–46. doi: 10.1002/acr2.11386 PMC8916578 34890478

[12] Bsteh G Bitschnau C Hegen H Auer M Di Pauli F Rommer P. Multiple sclerosis and COVID-19: How many are at risk? Eur J Neurol (2021) 28:3369–74. doi: 10.1111/ene.14555 PMC7537184 32978860

[13] Buljevac D Flach HZ Hop WC Hijdra D Laman JD Savelkoul HF. Prospective study on the relationship between infections and multiple sclerosis exacerbations. Brain (2002) 125:952–60. doi: 10.1093/brain/awf098 11960885

[14] Mailand MT Frederiksen JL. Vaccines and multiple sclerosis: a systematic review. J Neurol (2017) 264:1035–50. doi: 10.1007/s00415-016-8263-4 27604618

[15] Hernan MA Alonso A Hernandez-Diaz S. Tetanus vaccination and risk of multiple sclerosis: a systematic review. Neurology (2006) 67:212–5. doi: 10.1212/01.wnl.0000225079.51201.f9 16864810

[16] Farez MF Correale J. Yellow fever vaccination and increased relapse rate in travelers with multiple sclerosis. Arch Neurol (2011) 68:1267–71. doi: 10.1001/archneurol.2011.131 21670384

[17] Heinz FX Stiasny K. Distinguishing features of current COVID-19 vaccines: knowns and unknowns of antigen presentation and modes of action. NPJ Vaccines (2021) 6:104. doi: 10.1038/s41541-021-00369-6 34400651 PMC8368295

[18] Pardi N Hogan M Porter F Weissman D. mRNA vaccines — a new era in vaccinology. Nat Rev Drug Discovery (2018) 17:261–79. doi: 10.1038/nrd.2017.243 PMC5906799 29326426

[19] Meyer M Huang E Yuzhakov O Ramanathan P Ciaramella G Bukreyev A. Modified mRNA-based vaccines elicit robust immune responses and protect Guinea pigs from Ebola virus disease. J Infect Dis (2018) 217:451–5. doi: 10.1093/infdis/jix592 PMC5853918 29281112

[20] Pattnaik A Sahoo BR Pattnaik AK. Current status of zika virus vaccines: Successes and challenges. Vaccines (Basel) (2020) 8:266. doi: 10.3390/vaccines8020266 PMC7349928 32486368

[21] European Medicines Agency. Comirnaty . Available at: https://www.ema.europa.eu/en/medicines/human/EPAR/comirnaty (Accessed April 27, 2022).

[22] European Medicines Agency. Spikevax . Available at: https://www.ema.europa.eu/en/medicines/human/EPAR/spikevax (Accessed April 27, 2022).

[23] European Medicines Agency. Vaxzevria . Available at: https://www.ema.europa.eu/en/medicines/human/EPAR/vaxzevria-previously-covid-19-vaccine-astrazeneca (Accessed April 27, 2022).

[24] European Medicines Agency. COVID-19 vaccine janssen . Available at: https://www.ema.europa.eu/en/medicines/human/EPAR/covid-19-vaccine-janssen (Accessed April 27, 2022).

[25] European Medicines Agency. Nuvaxovid . Available at: https://www.ema.europa.eu/en/medicines/human/EPAR/nuvaxovid (Accessed April 27, 2022).

[26] COVID-19 vaccine tracker. Vaccine candidates in clinical trials . Available at: https://covid19.trackvaccines.org/vaccines/#progress-meter (Accessed June 17, 2022).

[27] COVID-19 vaccine tracker. Sinopharm (Beijing): Covilo . Available at: https://covid19.trackvaccines.org/vaccines/5/ (Accessed April 27, 2022).

[28] COVID-19 vaccine tracker. Sinovac: CoronaVac . Available at: https://covid19.trackvaccines.org/vaccines/7/ (Accessed April 27, 2022).

[29] COVID-19 vaccine tracker. Meissa vaccines inc: MV-014-212 . Available at: https://covid19.trackvaccines.org/vaccines/98/ (Accessed April 27, 2022).

[30] COVID-19 vaccine tracker. Inovio: INO-4800 . Available at: https://covid19.trackvaccines.org/vaccines/17/ (Accessed April 27, 2022).

[31] Achiron A Dolev M Menascu S Zohar DN Dreyer-Alster S Miron S. COVID-19 vaccination in patients with multiple sclerosis: What we have learnt by February 2021. Mult Scler (2021) 27:864–70. doi: 10.1177/13524585211003476 PMC8114441 33856242

[32] Allen-Philbey K Stennett A Begum T Johnson AC Dobson R Giovannoni G. Experience with the COVID-19 AstraZeneca vaccination in people with multiple sclerosis. Mult Scler Relat Disord (2021) 52:103028. doi: 10.1016/j.msard.2021.103028 34049216 PMC8129799

[33] Briggs FBS Mateen FJ Schmidt H Currie KM Siefers HM Crouthamel S. COVID-19 vaccination reactogenicity in persons with multiple sclerosis. Neurol Neuroimmunol Neuroinflamm (2021) 9 (1):e1104. doi: 10.1212/NXI.0000000000001104 34753828 PMC8579248

[34] Fragoso YD Gomes S Gonçalves MVM Mendes Junior E Oliveira BES Rocha CF. New relapse of multiple sclerosis and neuromyelitis optica as a potential adverse event of AstraZeneca AZD1222 vaccination for COVID-19. Mult Scler Relat Disord (2022) 57:103321. doi: 10.1016/j.msard.2021.103321 35158439 PMC8511887

[35] Nistri R Barbuti E Rinaldi V Tufano L Pozzilli V Ianniello A. Case report: Multiple sclerosis relapses after vaccination against SARS-CoV2: A series of clinical cases. Front Neurol (2021) 12:765954. doi: 10.3389/fneur.2021.765954 34744992 PMC8569136

[36] Khayat-Khoei M Bhattacharyya S Katz J Harrison D Tauhid S Bruso P. COVID-19 mRNA vaccination leading to CNS inflammation: a case series. J Neurol (2022) 269:1093–106. doi: 10.1007/s00415-021-10780-7 PMC8417681 34480607

[37] Maniscalco GT Manzo V Di Battista ME Salvatore S Moreggia O Scavone C. Severe multiple sclerosis relapse after COVID-19 vaccination: A case report. Front Neurol (2021) 12:721502. doi: 10.3389/fneur.2021.721502 34447349 PMC8382847

[38] National Multiple Sclerosis Society. Disease modifying therapy guidelines during COVID-19 . Available at: https://www.nationalmssociety.org/coronavirus-covid-19-information/multiple-sclerosis-and-coronavirus/ms-treatment-guidelines-during-coronavirus (Accessed April 29, 2022).

[39] MS international federation. COVID-19 vaccines and MS . Available at: https://www.msif.org/news/2020/02/10/the-coronavirus-and-ms-what-you-need-to-know/ (Accessed June 16, 2022).

[40] Reyes S Cunningham AL Kalincik T Havrdová EK Isobe N Pakpoor J. Update on the management of multiple sclerosis during the COVID-19 pandemic and post pandemic: An international consensus statement. J Neuroimmunol (2021) 357:577627. doi: 10.1016/j.jneuroim.2021.577627 34139567 PMC8183006

[41] Korsukewitz C Reddel SW Bar-Or A Wiendl H. Neurological immunotherapy in the era of COVID-19 - looking for consensus in the literature [published correction appears in nat rev neurol. 2020 Jul 22] Nat Rev Neurol (2020) 16 (9):493–505. doi: 10.1038/s41582-020-0385-8 PMC7341707 32641860

[42] Toscano S Chisari CG Patti F. Multiple sclerosis, COVID-19 and vaccines: Making the point. Neurol Ther (2021) 10:627–49. doi: 10.1007/s40120-021-00288-7 PMC8500471 34625925

[43] MS International Federation. MS, the coronavirus and vaccines – updated global advice . Available at: https://www.msif.org/news/2020/02/10/the-coronavirus-and-ms-what-you-need-to-know/ (Accessed April 29, 2022).

[44] Capuano R Donnarumma G Bisecco A Grimaldi E Conte M d'Ambrosio A. Humoral response to SARS-CoV-2 mRNA vaccine in patients with multiple sclerosis treated with natalizumab. Ther Adv Neurol Disord (2021) 14:17562864211038111. doi: 10.1177/17562864211038111 34413902 PMC8369851

[45] Achiron A Mandel M Dreyer-Alster S Harari G Magalashvili D Sonis P. Humoral immune response to COVID-19 mRNA vaccine in patients with multiple sclerosis treated with high-efficacy disease-modifying therapies. Ther Adv Neurol Disord SAGE Publ Ltd STM; (2021) 14:17562864211012836. doi: 10.1177/17562864211012835 PMC8072850 34035836

[46] Pitzalis M Idda ML Lodde V Loizedda A Lobina M Zoledziewska M. Effect of different disease-modifying therapies on humoral response to BNT162b2 vaccine in sardinian multiple sclerosis patients. Front Immunol (2021) 12:781843. doi: 10.3389/fimmu.2021.781843 34956211 PMC8697018

[47] Sabatino JJ Jr Mittl K Rowles WM McPolin K Rajan JV Laurie MT. Multiple sclerosis therapies differentially affect SARS-CoV-2 vaccine-induced antibody and T cell immunity and function. JCI Insight (2022) 7:e156978. doi: 10.1172/jci.insight.156978 35030101 PMC8876469

[48] Reyes S Ramsay M Ladhani S Amirthalingam G Singh N Cores C. Protecting people with multiple sclerosis through vaccination. Pract Neurol (2020) 20:435–45. doi: 10.1136/practneurol-2020-002527 32632038

[49] Sormani MP Inglese M Schiavetti I Carmisciano L Laroni A Lapucci C. Effect of SARS-CoV-2 mRNA vaccination in MS patients treated with disease modifying therapies. EBioMedicine (2021) 72:103581. doi: 10.1016/j.ebiom.2021.103581 34563483 PMC8456129

[50] Sadarangani M Marchant A Kollmann TR. Immunological mechanisms of vaccine-induced protection against COVID-19 in humans. Nat Rev Immunol (2021) 21:475–84. doi: 10.1038/s41577-021-00578-z PMC8246128 34211186

[51] Bhise V Dhib-Jalbut S. Potential risks and benefits of multiple sclerosis immune therapies in the COVID-19 era: Clinical and immunological perspectives. Neurother: J Am Soc Exp Neurother (2021) 18:244–51. doi: 10.1007/s13311-021-01008-7 PMC7853164 33533012

[52] Bar-Or A Calkwood JC Chognot C Evershed J Fox EJ Herman A. Effect of ocrelizumab on vaccine responses in patients with multiple sclerosis: The VELOCE study. Neurology (2020) 95:e1999–2008. doi: 10.1212/WNL.0000000000010380 PMC7843152 32727835

[53] Nojszewska M Kalinowska A Adamczyk-Sowa M Kułakowska A Bartosik-Psujek H. COVID-19 mRNA vaccines (Pfizer-BioNTech and moderna) in patients with multiple sclerosis: a statement by a working group convened by the section of multiple sclerosis and neuroimmunology of the polish neurological society. Neurol Neurochir Pol (2021) 55:8–11. doi: 10.5603/PJNNS.a2021.0016 33555604

[54] Gallo A Capuano R Donnarumma G Bisecco A Grimaldi E Conte M. Preliminary evidence of blunted humoral response to SARS-CoV-2 mRNA vaccine in multiple sclerosis patients treated with ocrelizumab. Neurol Sci (2021) 42:3523–6. doi: 10.1007/s10072-021-05397-7 PMC8203306 34128150

[55] Räuber S Korsen M Huntemann N Rolfes L Müntefering T Dobelmann V. Immune response to SARS-CoV-2 vaccination in relation to peripheral immune cell profiles among patients with multiple sclerosis receiving ocrelizumab. J Neurol Neurosurg Psychiatry (2022), 93 9:978–85. doi: 10.1136/jnnp-2021-328197 35193952 PMC8889453

[56] Brill L Rechtman A Zveik O Haham N Oiknine-Djian E Wolf DG. Humoral and T-cell response to SARS-CoV-2 vaccination in patients with multiple sclerosis treated with ocrelizumab. JAMA Neurol (2021) 78:1510–4. doi: 10.1001/jamaneurol.2021.3599 PMC8461553 34554197

[57] Gadani SP Reyes-Mantilla M Jank L Harris S Douglas M Smith MD. Discordant humoral and T cell immune responses to SARS-CoV-2 vaccination in people with multiple sclerosis on anti-CD20 therapy. EBioMedicine (2021) 73:103636. doi: 10.1016/j.ebiom.2021.103636 34666226 PMC8520057

[58] Yuzefpolskiy Y Morawski P Fahning M Speake C Lord S Chaudhary A. Cutting edge: Effect of disease-modifying therapies on SARS-CoV-2 vaccine-induced immune responses in multiple sclerosis patients. J Immunol (2022) 208:1519–24. doi: 10.4049/jimmunol.2101142 35288472

[59] Apostolidis SA Kakara M Painter MM Goel RR Mathew D Lenzi K. Cellular and humoral immune responses following SARS-CoV-2 mRNA vaccination in patients with multiple sclerosis on anti-CD20 therapy. Nat Med (2021) 27:1990–2001. doi: 10.1038/s41591-021-01507-2 34522051 PMC8604727

[60] Kornek B Leutmezer F Rommer PS Koblischke M Schneider L Haslacher H. B cell depletion and SARS-CoV-2 vaccine responses in neuroimmunologic patients. Ann Neurol (2022) 91:342–52. doi: 10.1002/ana.26309 PMC9011809 35067959

[61] Achiron A Mandel M Dreyer-Alster S Harari G Dolev M Menascu S. Humoral immune response in multiple sclerosis patients following PfizerBNT162b2 COVID19 vaccination: Up to 6 months cross-sectional study. J Neuroimmunol (2021) 361:577746. doi: 10.1016/j.jneuroim.2021.577746 34655991 PMC8500842

[62] Ciampi E Uribe-San-Martin R Soler B García L Guzman J Pelayo C. Safety and humoral response rate of inactivated and mRNA vaccines against SARS-CoV-2 in patients with multiple sclerosis. Mult Scler Relat Disord (2022) 59:103690. doi: 10.1016/j.msard.2022.103690 35182880 PMC8842089

[63] Centonze D Rocca MA Gasperini C Kappos L Hartung HP Magyari M. Disease-modifying therapies and SARS-CoV-2 vaccination in multiple sclerosis: an expert consensus. J Neurol (2021) 268:3961–8. doi: 10.1007/s00415-021-10545-2 PMC8038920 33844056

[64] Rieckmann P Centonze D Giovannoni G Hua LH Oreja-Guevara C Selchen D. Expert opinion on COVID-19 vaccination and the use of cladribine tablets in clinical practice. Ther Adv Neurol Disord (2021) 14:17562864211058298. doi: 10.1177/17562864211058298 34899987 PMC8655448

[65] Yamout BI Rieckman P Centonze D Giovannoni G Hua LH Oreja-Guevara C. Expert opinion on COVID-19 vaccination and the use of cladribine tablets. Multiple Sclerosis Related Disord (2022) 59:103621. doi: 10.1016/j.msard.2022.103621

[66] Greco R Alexander T Burman J Del Papa N de Vries-Bouwstra J Farge D. Hematopoietic stem cell transplantation for autoimmune diseases in the time of COVID-19: EBMT guidelines and recommendations. Bone Marrow Transplant (2021) 56:1493–508. doi: 10.1038/s41409-021-01326-6 PMC8143059 34031556

[67] National Multiple Sclerosis Society. Preferred COVID-19 vaccines for those living with MS . Available at: https://www.nationalmssociety.org/coronavirus-covid-19-information/multiple-sclerosis-and-coronavirus/covid-19-vaccine-guidance#section-4 (Accessed April 29, 2022).

[68] Beatty AL Peyser ND Butcher XE Cocohoba JM Lin F Olgin JE. Analysis of COVID-19 vaccine type and adverse effects following vaccination. JAMA Netw Open (2021) 4:e2140364. doi: 10.1001/jamanetworkopen.2021.40364 34935921 PMC8696570

[69] European Medicines Agency. Comirnaty and spikevax: possible link to very rare cases of myocarditis and pericarditis . Available at: https://www.ema.europa.eu/en/news/comirnaty-spikevax-possible-link-very-rare-cases-myocarditis-pericarditis (Accessed April 29, 2022).

[70] Oster ME Shay DK Su JR Gee J Creech CB Broder KR. Myocarditis cases reported after mRNA-based COVID-19 vaccination in the US from December 2020 to august 2021. JAMA (2022) 327:331–40. doi: 10.1001/jama.2021.24110 PMC8790664 35076665

[71] European Medicines Agency. AstraZeneca’s COVID-19 vaccine: EMA finds possible link to very rare cases of unusual blood clots with low blood platelets . Available at: https://www.ema.europa.eu/en/news/astrazenecas-covid-19-vaccine-ema-finds-possible-link-very-rare-cases-unusual-blood-clots-low-blood (Accessed April 29, 2022).

[72] Badrawi N Kumar N Albastaki U. Post COVID-19 vaccination neuromyelitis optica spectrum disorder: Case report & MRI findings. Radiol Case Rep (2021) 16:3864–7. doi: 10.1016/j.radcr.2021.09.033 PMC8512112 34659602

[73] Levin EG Lustig Y Cohen C Fluss R Indenbaum V Amit S. Waning immune humoral response to BNT162b2 covid-19 vaccine over 6 months. N Engl J Med (2021) 385(24):e84. doi: 10.1056/NEJMoa2114583 34614326 PMC8522797

[74] Menni C May A Polidori L Louca P Wolf J Capdevila J. COVID-19 vaccine waning and effectiveness and side-effects of boosters: a prospective community study from the ZOE COVID study. Lancet Infect Dis (2022) S1473-3099(22):00146–3. doi: 10.1016/S1473-3099(22)00146-3 PMC8993156 35405090

[75] Andrews N Stowe J Kirsebom F Toffa S Sachdeva R Gower C. Effectiveness of COVID-19 booster vaccines against COVID-19-related symptoms, hospitalization and death in England. Nat Med (2022) 28:831–7. doi: 10.1038/s41591-022-01699-1 PMC9018410 35045566

[76] Jr MED Kitchin N Xu X Dychter SS Lockhart S Gurtman A. Clinical trial group. safety and efficacy of a third dose of BNT162b2 covid-19 vaccine. N Engl J Med (2022) 386:1910–21. doi: 10.1056/NEJMoa2200674 PMC9006787 35320659

[77] Dreyer-Alster S Menascu S Mandel M Shirbint E Magalashvili D Dolev M. COVID-19 vaccination in patients with multiple sclerosis: Safety and humoral efficacy of the third booster dose. J Neurol Sci (2022) 434:120155. doi: 10.1016/j.jns.2022.120155 35091386 PMC8779784

[78] König M Torgauten HM Tran TT Holmøy Y Torgils Vaage J. Lund-Johansen F, et al. immunogenicity and safety of a third SARS-CoV-2 vaccine dose in patients with multiple sclerosis and weak immune response after COVID-19 vaccination. JAMA Neurol (2022) 79:307–9. doi: 10.1001/jamaneurol.2021.5109 PMC8787678 35072702

[79] Sormani MP Schiavetti I Inglese M Carmisciano L Laroni A Lapucci C. Breakthrough SARS-CoV-2 infections after COVID-19 mRNA vaccination in MS patients on disease modifying therapies during the delta and the omicron waves in Italy. EBioMedicine (2022) 80:104042. doi: 10.1016/j.ebiom.2022.104042 35526306 PMC9069178

[80] Caillard S Thaunat O Benotmane I Masset C Blancho G. Antibody response to a fourth messenger RNA COVID-19 vaccine dose in kidney transplant recipients: A case series. Ann Intern Med (2022) 175:455–6. doi: 10.7326/L21-0598 PMC8754215 35007148

[81] Bar-On YM Goldberg Y Mandel M Bodenheimer O Amir O Freedman L. Protection by a fourth dose of BNT162b2 against omicron in Israel. N Engl J Med (2022) 386:1712–20. doi: 10.1056/NEJMoa2201570 PMC9006780 35381126

[82] Yap SM Al Hinai M Gaughan M Callanan I Kearney H Tubridy N. Mult scler relat disord. (2021) 56:103236. doi: 10.1016/j.msard.2021.103236 34507240 PMC8411656

[83] Abbasi N Ghadiri F Moghadasi AN Azimi A Navardi S Heidari H. COVID-19 vaccine hesitancy in Iranian patients with multiple sclerosis. Mult Scler Relat Disord (2022) 60:103723. doi: 10.1016/j.msard.2022.103723 35276452 PMC8896865

[84] Uhr L Mateen FJ. COVID-19 vaccine hesitancy in multiple sclerosis: A cross-sectional survey. Mult Scler (2021) 28 (7) 1072–80. doi: 10.1177/13524585211030647 34313513

[85] Winkelmann A Loebermann M Barnett M Hartung HP Zettl UK. Vaccination and immunotherapies in neuroimmunological diseases. Nat Rev Neurol (2022) 18 (5):289–306. doi: 10.1038/s41582-022-00646-5 35388213 PMC8985568

